# Relationship between serum carotenoids and telomere length in overweight or obese individuals

**DOI:** 10.3389/fnut.2024.1479994

**Published:** 2024-11-22

**Authors:** Jiang Wang, Fayi Xie, Wan Zhu, Dongmei Ye, Yi Xiao, Mengxia Shi, Rui Zeng, Jiahui Bian, Xiao Xu, Lihuan Chen, Aizhang Zhu, Ke Zhu, Tenghui Fan, Bin Liu, Liyan Xiao, Xiaoming Zhang

**Affiliations:** ^1^School of Basic Medicine, Jinggangshan University, Ji’an, China; ^2^Online Collaborative Research Center for Evidence-Based Medicine Ministry of Education, Jinggangshan University, Ji’an, China; ^3^School of Clinical Medicine, Jinggangshan University, Ji’an, China; ^4^School of Mathematics and Physics, Jinggangshan University, Ji’an, China; ^5^State Key Laboratory of Cardiovascular Diseases, Shanghai East Hospital, School of Medicine, Tongji University, Shanghai, China; ^6^School of Chinese Medicine, Jinggangshan University, Ji’an, China; ^7^School of Foreign Languages, Jinggangshan University, Ji’an, China; ^8^Department of Emergency, The People’s Hospital of Baoan Shenzhen, Shenzhen, China

**Keywords:** *β*-carotene, carotenoids, NHANES, obesity, telomeres

## Abstract

**Background:**

Previous researches have demonstrated an association between carotenoids and elongated telomeres. Nonetheless, there is scant scientific evidence examining this relationship in individuals who are overweight or obese, a demographic more predisposed to accelerated aging. This study aims to elucidate the correlation between serum carotenoid concentrations and telomere length within this population group.

**Methods:**

Data were sourced from the 2001–2002 National Health and Nutrition Examination Survey, encompassing 2,353 overweight or obese participants. The levels of *α*-carotene, *β*-carotene (both trans and cis isomers), *β*-cryptoxanthin, lutein/zeaxanthin, and trans-lycopene were quantified via high-performance liquid chromatography. Telomere length was assessed using quantitative polymerase chain reaction.

**Results:**

Following adjustment for potential confounders, telomere length exhibited an increase of 1.83 base pairs (bp) per unit elevation in *β*-carotene levels (*β* = 1.83; 95% CI: 0.48, 3.18). Within the fully adjusted model, telomere length incremented by 1.7 bp per unit increase in serum *β*-carotene among overweight individuals (*β* = 1.7; 95% CI: 0.1, 3.3), and by 2.6 bp per unit increase among obese individuals (*β* = 2.6; 95% CI: 0.1, 5.0). Furthermore, restricted cubic spline analysis revealed a linear relationship between *β*-carotene levels and telomere length, whereas a non-linear association was observed between *β*-cryptoxanthin levels and telomere length.

**Conclusion:**

This investigation indicates that higher serum *β*-carotene concentrations are linked with extended telomere length in overweight and obese populations in the United States. These findings warrant further validation through prospective studies.

## 1 Introduction

Serum carotenoids are well-recognized natural antioxidants, with over 95% of carotenoids in human blood circulation primarily consisting of *β*-carotene, *α*-carotene, *β*-cryptoxanthin, lutein/zeaxanthin, and lycopene. These carotenoids exhibit potent antioxidant properties, mitigating damage induced by reactive oxygen species and inhibiting lipid peroxidation (1). Additionally, carotenoids are involved in cellular signaling pathways associated with inflammation and oxidative stress (OS), thereby exerting a modulatory effect on both OS and inflammation (2).

Telomeres, located at the termini of linear chromosomes, are composed of thousands of TTAGGG nucleotide sequence repeats, serving to protect chromosome ends from deterioration and preventing chromosomal fusion (3). Genetic factors play a crucial role in determining telomere length. Concurrently, the preservation of telomere length is integral to genomic stability and aging. Throughout an individual’s lifespan, telomeres progressively shorten with each cell division (4). In essence, various environmental factors impacting genomic stability, aging, oxidative and inflammatory responses—such as diet, smoking, obesity, and physical activity—contribute to alterations in telomere length (5, 6).

Despite the established correlation between serum carotenoids and telomere length, there remains a paucity of information regarding this relationship in overweight or obese individuals. To our knowledge, this is the inaugural study exploring the association between serum carotenoids and telomere length in overweight or obese populations within the United States. Over recent decades, the prevalence of obesity has surged globally, attributed to shifts in dietary habits and lifestyle choices (7). Obesity, characterized by the excessive accumulation of adipose tissue (AT), involves the release of adipokines from AT, which regulate various biological processes such as inflammation, insulin resistance, and glucose and lipid metabolism, thereby contributing to the pathogenesis of obesity-related diseases (8).

Therefore, the objective of this study is to investigate the relationship between serum carotenoid concentrations and telomere length among individuals classified as overweight or obese, using data from the 2001/2002 cycle of the National Health and Nutrition Examination Survey (NHANES). Given the antioxidant properties of carotenoids, we hypothesize that higher carotenoid levels may attenuate telomere shortening in the study population.

## 2 Methods

### 2.1 Study population

The National Health and Nutrition Examination Survey (NHANES) is an extensive research initiative designed to evaluate the health and nutritional status of adults and children in the United States. NHANES has received formal approval from the US Centers for Disease Control and Prevention’s Research Ethics Review Board, with written informed consent obtained from all study participants. The datasets generated and analyzed in this study are publicly accessible on the NHANES official website.^1^

**Figure 1:**
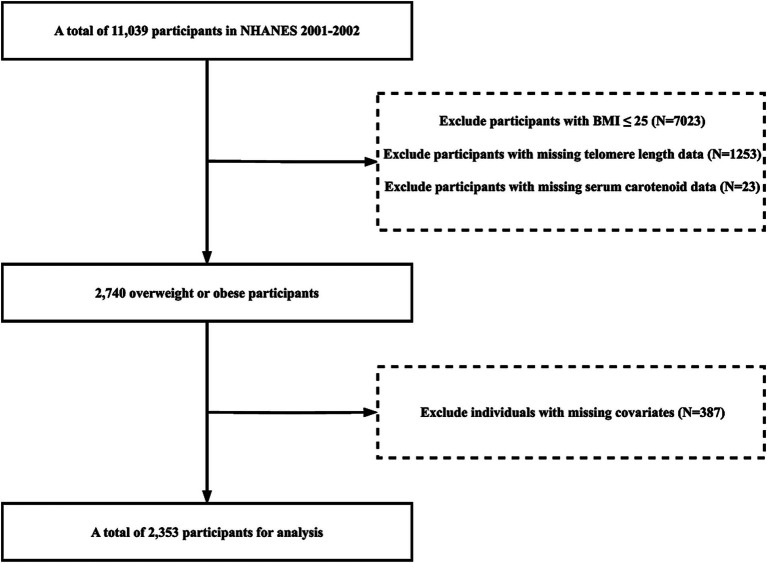
Flowchart of the study population.

Our study population was derived from the 2001–2002 NHANES database. Initially, we screened 11,039 participants to identify overweight and obese individuals, excluding those with a BMI ≤ 25 kg/m^2^ (N = 7,023). Further exclusions were made for individuals with missing data on telomere length, carotenoid levels, education, poverty income ratio (PIR), physical activity, energy intake, congestive heart failure, cancer or malignancy, hypertension, smoking, and alcohol consumption. 2,353 participants were included in our analysis (Figure 1).

### 2.2 Assessment of serum carotenoid levels

Serum specimens for carotenoid measurement were processed, stored, and shipped to the National Center for Disease Control and Prevention’s Department of Laboratory Sciences for analysis. The primary carotenoids measured in NHANES 2001–2002 were *α*-carotene, trans-*β*-carotene, cis-*β*-carotene, *β*-cryptoxanthin, combined lutein/zeaxanthin, and trans-lycopene. These measurements were conducted using high-performance liquid chromatography with photodiode array detection. Detailed laboratory procedures and quality control methods for serum carotenoid measurements are available elsewhere (9). The serum concentrations of total carotenoids were calculated by summing the concentrations of the five carotenoids listed above.

### 2.3 Assessment of telomere length

For DNA analysis, whole blood samples were collected from participants, and quantitative polymerase chain reaction (qPCR) was performed to determine telomere length (T/S ratio) related to standard reference DNA in Dr. Elizabeth Blackburn’s laboratory in San Francisco, *CA.* Further details on telomere length determination are available on the laboratory section’s website.^2^ The inter-assay coefficient of variation was 6.5%. A T/S ratio to base pair conversion was utilized, with the conversion formula being 3,274 + 2413*(T/S). Rigorous quality control reviews were conducted by the Centers for Disease Control and Prevention before linking telomere data to the NHANES 2001–2002 public data files.

### 2.4 Assessment of covariates

Demographic information included age, sex, education, race, poverty income ratio (PIR), BMI, and energy intake. Questionnaire data covered physical activity, smoking status, and alcohol consumption status. BMI was calculated as weight/height^2^ (kg/m^2^). Ethnicity was categorized as non-Hispanic white, non-Hispanic Black, Mexican American, other Hispanic, or other races. Physical activity levels were classified into no aerobic activity, low-level exercise, moderate-level exercise, and high-level exercise. The NHANES definitions were used to classify physical activity levels, ranging from predominantly sedentary to high-load activities. Smokers were defined as individuals who had smoked more than one hundred cigarettes in their lifetime, while drinkers were participants who consumed at least 12 alcoholic drinks of any type in a given year.

Medical history variables included hypertension, defined as mean systolic blood pressure ≥ 140 mmHg, mean diastolic blood pressure ≥ 90 mmHg, or self-reported hypertension. Data on congestive heart failure and cancer or malignancy were obtained through self-report questionnaires.

### 2.5 Statistical analysis

Continuous variables were presented as mean ± standard deviation, while categorical variables were expressed as counts (percentages). Serum total carotenoids were divided into quartiles. Baseline characteristics across different quartiles were assessed using chi-square tests and analysis of variance (Table 1). Generalized linear models were constructed to evaluate the relationship between serum carotenoids and telomere length in overweight or obese participants. Logistic regression models were used to assess the relationship between each quartile of serum carotenoids and their lowest quartile, with linear trends calculated by treating carotenoid quartiles as continuous variables (Table 2). Similar analyses were conducted in non-overweight and non-obese individuals (Supplementary Table S1). Further regression analyses were performed by categorizing participants into overweight (BMI < 30 kg/m^2^) and obese (BMI ≥ 30 kg/m^2^) subgroups based on obesity thresholds (Table 3).

**Table 1:** Baseline characteristics of participants.

	Q1	Q2	Q3	Q4	*p*-value
*N*	588	587	587	591	
Age, years	48.366 ± 17.263	47.329 ± 17.217	49.543 ± 17.692	52.252 ± 17.683	<0.001
BMI, kg/m^2^	32.733 ± 6.732	31.450 ± 5.525	30.530 ± 4.593	29.514 ± 3.579	<0.001
PIR	2.500 ± 1.582	2.684 ± 1.593	2.965 ± 1.615	2.964 ± 1.620	<0.001
Energy, kcal	2127.995 ± 1088.242	2156.126 ± 924.619	2154.814 ± 1017.206	2120.315 ± 923.328	0.527
Telomere length, bp	5764.713 ± 628.233	5798.925 ± 596.983	5803.733 ± 615.959	5775.862 ± 576.058	0.460
Alpha-carotene, ug/dl	1.422 ± 1.149	2.363 ± 1.479	3.841 ± 2.651	7.565 ± 6.822	<0.001
Beta-carotene (trans + cis), ug/dl	6.531 ± 3.416	11.306 ± 5.459	17.161 ± 7.709	36.239 ± 25.162	<0.001
Beta-cryptoxanthin, ug/dl	4.484 ± 2.356	7.370 ± 3.388	10.630 ± 5.475	18.443 ± 12.154	<0.001
Combined lutein/zeaxanthin, ug/dl	9.413 ± 3.509	13.034 ± 4.491	16.875 ± 5.994	23.693 ± 9.861	<0.001
Trans-lycopene, ug/dl	13.593 ± 6.094	20.598 ± 7.583	25.219 ± 9.436	29.424 ± 12.733	<0.001
Sex					0.757
Male	297 (50.510%)	289 (49.233%)	300 (51.107%)	285 (48.223%)	
Female	291 (49.490%)	298 (50.767%)	287 (48.893%)	306 (51.777%)	
Education					<0.001
Less Than 9th Grade	67 (11.395%)	63 (10.733%)	76 (12.947%)	106 (17.936%)	
9-11th Grade	119 (20.238%)	106 (18.058%)	79 (13.458%)	86 (14.552%)	
High School Grad	156 (26.531%)	160 (27.257%)	142 (24.191%)	104 (17.597%)	
Some College	164 (27.891%)	157 (26.746%)	164 (27.939%)	141 (23.858%)	
College Graduate	82 (13.946%)	101 (17.206%)	126 (21.465%)	154 (26.058%)	
Race					<0.001
Mexican American	99 (16.837%)	125 (21.295%)	121 (20.613%)	186 (31.472%)	
Other Hispanic	28 (4.762%)	22 (3.748%)	24 (4.089%)	18 (3.046%)	
Non-Hispanic White	343 (58.333%)	307 (52.300%)	307 (52.300%)	284 (48.054%)	
Non-Hispanic Black	111 (18.878%)	123 (20.954%)	108 (18.399%)	92 (15.567%)	
Other Race	7 (1.190%)	10 (1.704%)	27 (4.600%)	11 (1.861%)	
Physical activity					<0.001
no aerobic activity	176 (29.932%)	165 (28.109%)	139 (23.680%)	114 (19.289%)	
low level exercise	299 (50.850%)	286 (48.722%)	327 (55.707%)	339 (57.360%)	
moderate level exercise	80 (13.605%)	90 (15.332%)	78 (13.288%)	104 (17.597%)	
high level exercise	33 (5.612%)	46 (7.836%)	43 (7.325%)	34 (5.753%)	
Congestive heart failure					0.029
Yes	28 (4.762%)	18 (3.066%)	19 (3.237%)	10 (1.692%)	
No	560 (95.238%)	569 (96.934%)	568 (96.763%)	581 (98.308%)	
Cancer or malignancy					0.910
Yes	56 (9.524%)	53 (9.029%)	50 (8.518%)	50 (8.460%)	
No	532 (90.476%)	534 (90.971%)	537 (91.482%)	541 (91.540%)	
Hypertension					0.057
No	298 (50.680%)	337 (57.411%)	338 (57.581%)	321 (54.315%)	
Yes	290 (49.320%)	250 (42.589%)	249 (42.419%)	270 (45.685%)	
Smoking					<0.001
Yes	344 (58.503%)	310 (52.811%)	260 (44.293%)	234 (39.594%)	
No	244 (41.497%)	277 (47.189%)	327 (55.707%)	357 (60.406%)	
Drinking					0.302
Yes	415 (70.578%)	387 (65.928%)	389 (66.269%)	402 (68.020%)	
No	173 (29.422%)	200 (34.072%)	198 (33.731%)	189 (31.980%)	

BMI, Body mass index; PIR, Poverty income ratio; In case of continuous variables, Kruskal Wallis rank sum test was used, and if the theoretical number of count variables was < 10, Fisher exact probability test was used.

**Table 2:** Relationship between serum carotenoids and telomere length.

Exposure	Model I	Model II	Model III
	*β* 95% CI *p* value	*β* 95% CI p value	*β* 95% CI *p* value
Alpha-carotene	3.91 (−1.12, 8.94) 0.1273	4.29 (−0.90, 9.48) 0.1051	4.62 (−0.63, 9.86) 0.0845
Quartile of alpha-carotene			
Q1	Reference	Reference	Reference
Q2	−14.07 (−77.57, 49.43) 0.6641	0.72 (−63.98, 65.43) 0.9825	4.48 (−60.34, 69.31) 0.8922
Q3	29.81 (−35.20, 94.82) 0.3688	48.71 (−19.36, 116.78) 0.1609	56.46 (−12.01, 124.94) 0.1062
Q4	19.18 (−45.72, 84.08) 0.5625	38.49 (−30.95, 107.94) 0.2774	44.13 (−26.14, 114.40) 0.2185
P for trend	0.3194	0.1448	0.1056
Beta-carotene (trans + cis)	1.68 (0.37, 2.98) 0.0118	1.69 (0.35, 3.03) 0.0138	1.83 (0.48, 3.18) **0.0079**
Quartile of Beta-carotene (trans + cis)			
Q1	Reference	Reference	Reference
Q2	26.35 (−36.51, 89.21) 0.4113	26.65 (−36.40, 89.71) 0.4075	33.34 (−29.79, 96.48) 0.3008
Q3	80.73 (16.87, 144.59) 0.0133	76.15 (10.99, 141.32) 0.0221	85.21 (19.80, 150.61) **0.0107**
Q4	61.22 (−3.90, 126.34) 0.0655	64.80 (−2.78, 132.39) 0.0603	73.01 (5.18, 140.84) **0.0350**
P for trend	**0.0250**	**0.0280**	**0.0153**
Beta-cryptoxanthin	0.19 (−2.36, 2.74) 0.8826	1.77 (−1.04, 4.58) 0.2165	1.93 (−0.90, 4.76) 0.1815
Quartile of Beta-cryptoxanthin			
Q1	Reference	Reference	Reference
Q2	62.18 (−0.64, 125.01) 0.0525	53.39 (−9.73, 116.52) 0.0975	58.29 (−4.85, 121.44) 0.0705
Q3	74.79 (11.96, 137.62) 0.0197	73.99 (9.41, 138.57) 0.0248	82.22 (17.40, 147.04) **0.0130**
Q4	51.95 (−10.82, 114.73) 0.1049	83.64 (14.28, 153.00) 0.0182	92.36 (22.37, 162.35) **0.0098**
P for trend	0.0979	**0.0146**	**0.0073**
Combined lutein/zeaxanthin	1.85 (−0.85, 4.55) 0.1793	1.38 (−1.44, 4.19) 0.3379	1.32 (−1.50, 4.14) 0.3588
Quartile of Combined lutein/zeaxanthin			
Q1	Reference	Reference	Reference
Q2	20.81 (−42.18, 83.79) 0.5174	13.58 (−49.78, 76.95) 0.6744	16.46 (−46.90, 79.83) 0.6106
Q3	−1.79 (−65.17, 61.59) 0.9559	−13.94 (−78.58, 50.70) 0.6726	−11.09 (−75.85, 53.66) 0.7371
Q4	26.55 (−37.17, 90.27) 0.4142	15.07 (−51.63, 81.76) 0.6580	14.96 (−51.82, 81.74) 0.6606
P for trend	0.5799	0.8752	0.8785
Trans-lycopene	2.05 (−0.04, 4.15) 0.0547	1.41 (−0.70, 3.53) 0.1900	1.38 (−0.73, 3.50) 0.2001
Quartile of Trans-lycopene			
Q1	Reference	Reference	Reference
Q2	−6.54 (−70.26, 57.19) 0.8407	−2.41 (−66.00, 61.18) 0.9407	−1.59 (−65.22, 62.04) 0.9610
Q3	47.19 (−16.91, 111.30) 0.1492	40.35 (−23.70, 104.40) 0.2170	40.45 (−23.70, 104.60) 0.2166
Q4	59.52 (−5.69, 124.73) 0.0738	46.36 (−19.39, 112.11) 0.1671	44.97 (−20.88, 110.82) 0.1809
P for trend	0.0260	0.0835	0.0924

Model I adjust for: Age; Sex. Model II adjust for: Age; Sex; Education; Race; PIR; BMI; Physical activity; Energy. Model III adjust for: Age; Sex; Education; Race; PIR; BMI; Physical activity; Energy; Congestive heart failure; Cancer or malignancy; Hypertension; Smoking; Drinking. Bold indicates significant statistical test value.

**Table 3:** Relationship between serum *β*-carotene and telomere length in overweight and obese people.

Beta-carotene (trans + cis)	Model I	Model II	Model III
BMI	*β* (95% CI) *p*- value	*β* (95% CI) *p*- value	*β* (95% CI) *p*- value
BMI ≤ 30	1.4 (−0.1, 3.0) 0.074	1.6 (−0.1, 3.2) 0.059	1.7 (0.1, 3.3) **0.042**
BMI > 30	2.0 (−0.4, 4.4) 0.107	2.4 (−0.1, 4.8) 0.058	2.6 (0.1, 5.0) **0.042**
Total	1.5 (0.2, 2.9) **0.022**	1.7 (0.4, 3.1) **0.010**	1.9 (0.5, 3.2) **0.006**

Model I adjust for: Age; Sex. Model II adjust for: Age; Sex; Education; Race; PIR; Physical activity; Energy. Model III adjust for: Age; Sex; Education; Race; PIR; Physical activity; Energy; Congestive heart failure; Cancer or malignancy; Hypertension; Smoking; Drinking. Bold indicates significant statistical test value.

**Figure 2:**
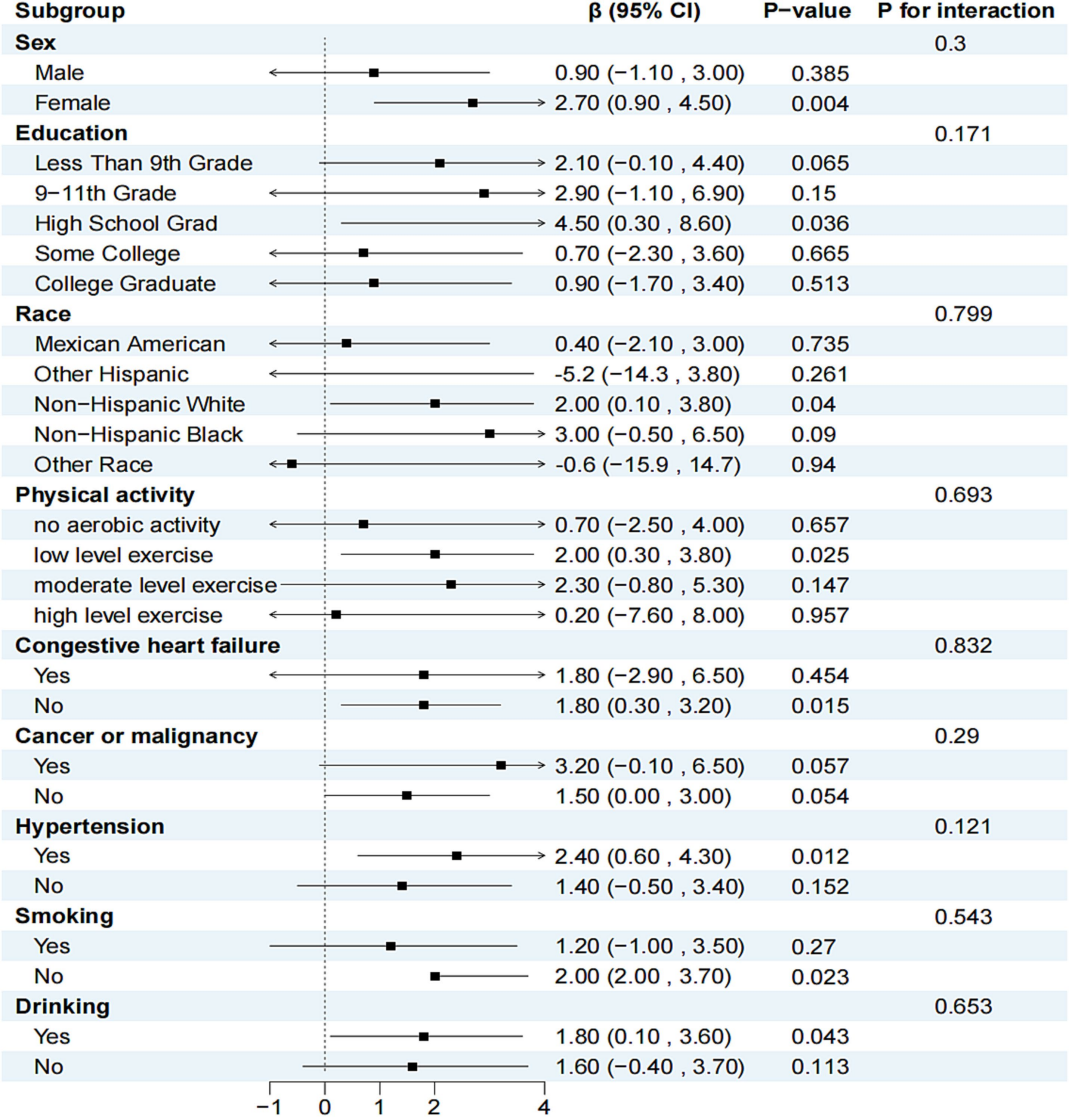
Relationship between serum *β*-carotene and telomere length in different subgroups. In addition to the stratification variables themselves, sex, education, race, physical activity, congestive heart failure, cancer or malignancy, hypertension, smoking and drinking were adjusted.

**Figure 3:**
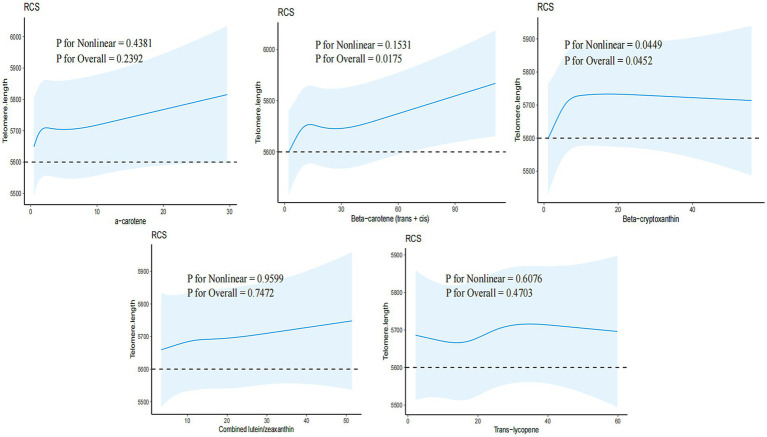
Association between serum *β*-carotene and telomere length in overweight and obese people. Adjusted for age, sex, education, race, PIR, BMI, physical activity, energy, congestive heart failure, cancer or malignancy, hypertension, smoking, and drinking.

Three models were employed to adjust for potential confounders identified in previous studies (10). Model I was adjusted for sex and age, Model II included additional demographic characteristics such as education, race, PIR, BMI, physical activity, and energy intake, and Model III further adjusted for medical history variables including congestive heart failure, cancer or malignancy, hypertension, smoking, and alcohol consumption. Stratified analyses were conducted to determine the relationship between serum carotenoids and telomere length across various subgroups based on sex, education, race, physical activity, congestive heart failure, cancer or malignancy, hypertension, smoking, and alcohol consumption (Figure 2). Lastly, a restricted cubic spline model with five nodes was utilized to examine the relationship between each serum carotenoid and telomere length (Figure 3).

All analyses were performed using a two-sided significance level (*p* < 0.05) with the statistical software packages R^3^ and Empower Stats.^4^

## 3 Results

### 3.1 Baseline characteristics of participants

The baseline characteristics of the 2,353 participants included in this study are presented in Table 1. The mean age of the subjects was 49.4 ± 17.6 years, with 1,171 males (49.8%). Participants in the highest serum total carotenoid group (Q4) were more likely to be female, older, and had a higher proportion of college graduates or higher education levels. This group also tended toward lower or moderate physical activity compared to other groups, had a lower mean BMI, and consumed less energy. Additionally, they were less likely to smoke or have conditions such as congestive heart failure.

### 3.2 Relationship between serum carotenoids and telomere length

The relationship between serum carotenoids and telomere length is detailed in Table 2. After multivariate adjustment, a significant relationship was observed between *β*-carotene (trans + cis) and telomere length, whereas no association was found with *α*-carotene, *β*-cryptoxanthin, lutein/zeaxanthin, and trans-lycopene. For continuous carotenoid levels, telomere length increased by 1.83 base pairs (bp) per unit increase in *β*-carotene levels (*β* = 1.83; 95% CI: 0.48, 3.18). When carotenoid levels were divided into quartiles, a significant positive correlation was found between the highest quartile and telomere length compared with the lowest quartile for *β*-carotene (OR = 73.1; 95% CI: 5.18, 140.84) and *β*-cryptoxanthin (OR = 92.36; 95% CI: 22.37, 162.35). Across all models, trend tests indicated statistically significant associations for *β*-carotene (*p* for trend <0.05). No such association was observed in non-overweight and non-obese individuals, as shown in Supplementary Table S1. In the fully adjusted model, telomere length increased by 1.7 bp per unit increase in serum *β*-carotene in overweight individuals (*β* = 1.7; 95% CI: 0.1, 3.3), and by 2.6 bp per unit increase in obese individuals (*β* = 2.6; 95% CI: 0.1, 5.0), as shown in Table 3.

### 3.3 Subgroup analysis

The relationship between serum *β*-carotene and telomere length within subgroups is shown in Figure 2. Subgroups were stratified by sex, education, ethnicity, physical activity, congestive heart failure, cancer or malignancy, hypertension, smoking, and alcohol consumption. After adjusting for variables other than the stratification variable itself, no significant interaction was found between *β*-carotene levels and potential confounders of telomere length (*p* > 0.05 for each interaction). Supplementary Tables S2–S5 present stratified analyses and interactions between the other four carotenoids and telomere length, with results similar to those for *β*-carotene.

### 3.4 Restricted cubic spline model

The dose–response relationship between carotenoid levels and telomere length is illustrated in Figure 3. No linear deviation from telomere length was observed for *β*-carotene (*p* for Nonlinear = 0.1531; *p* for Overall = 0.0175). However, a non-linear relationship was detected between *β*-cryptoxanthin and telomere length (*p* for Nonlinear = 0.0449; *p* for Overall = 0.0452), with a significant relationship below the threshold of 17.6 μg/dL. No nonlinear relationship was observed for the other three carotenoids.

## 4 Discussion

Our study found that increasing serum carotenoid levels were significantly associated with longer telomere lengths in overweight or obese U.S. populations. Specifically, *β*-carotene showed a linear correlation with telomere length, while *β*-cryptoxanthin showed a non-linear correlation. The other three carotenoids were not statistically significant. The increase in carotenoid levels had a more significant effect on telomere length in obese individuals compared to overweight individuals. Notably, no such relationship was found between carotenoids and telomere length in non-overweight or non-obese individuals.

Previous studies have shown a significant positive relationship between telomere length and self-reported high dietary intake of vegetables and *β*-carotene (11), particularly in women not using multivitamins (12). Serum carotenoid levels have also been highlighted as objective markers of dietary intake. A study from Austria indicated that higher plasma concentrations of lutein, zeaxanthin, and vitamin C were associated with longer leukocyte telomere length in normal older adults, suggesting a protective role for these vitamins in telomere maintenance (3). Similarly, an increase in blood carotenoid levels was significantly associated with longer leukocyte telomeres in 3,660 adults from NHANES (10). And this result was replicated in a larger cohort, serum carotenoids generally showed a positive correlation with leukocyte telomere length (13).

However, most previous studies have focused on the general adult population, neglecting groups more prone to accelerated aging, such as overweight or obese individuals (14). This longitudinal study highlights the significant finding that telomere shortening begins at a remarkably early age in children with obesity (15). This aligns with the understanding that obesity reduces telomere length by persistently affecting systemic inflammation and redox homeostasis (16). The urgent need for preventive measures and early interventions is emphasized to mitigate the long-term health consequences of obesity on telomere dynamics and associated metabolic disorders. For example, obese mice have shown reduced telomere length in oocytes and embryos (17), and overweight and obese children have significantly shorter telomeres compared to children with normal BMI (18). A collaborative cross-sectional meta-analysis of 87 observational studies also demonstrated that higher BMI is associated with shorter telomeres, particularly in young adults (19). Thus, maintaining a healthy body weight is crucial to delay telomere shortening and the development of related diseases. Additionally, a meta-analysis has demonstrated that psychological stress is linked to a reduction in telomere length (16), with high levels correlating with chronic diseases such as obesity and abdominal fat accumulation (20). Furthermore, beyond varying stress levels, socioeconomic status also influences telomere length. A cohort study from FFCWS identified poverty as a predictor of changes in telomere length among women (21). Moreover, certain stressors unique to women may further exacerbate this effect (22).

Mechanistically, increases in oxidative stress and chronic inflammation are key contributors to telomere shortening (23). Reactive oxygen species from oxidative stress can cause breaks in DNA and interfere with the replication of telomeric repeats, leading to an increased rate of telomere shortening. Chronic inflammation increases inflammatory mediators, which also promote telomere shortening. Conversely, telomere shortening in leukocytes leads to decreased immune function and increased secretion of pro-inflammatory factors (24, 25), creating a vicious cycle (26). Obesity exacerbates this cycle by increasing oxidative stress and chronic inflammation (16), potentially due to adipocyte proliferation and hypertrophy leading to adipose tissue hypoxia (27). Therefore, obese individuals may have shorter somatic telomere lengths and are more susceptible to premature aging and reduced cell lifespan (28–30). Our findings support this trend, with lower BMI associated with longer telomeres across all populations included.

In this study, serum *β*-carotene levels were significantly associated with longer telomeres, while *β*-cryptoxanthin showed no significant relationship beyond a certain concentration. *α*-carotene, lutein/zeaxanthin, and trans-lycopene were not statistically significant. This difference may be due to the study population size, statistical methods, and choice of confounding variables. Further carefully designed studies are needed to assess the effects of these carotenoids on telomeres (10).

Despite these differences, carotenoids still play a significant role in protecting against telomere loss. Tocopherol (vitamin E) and *β*-carotene work synergistically to quench reactive oxygen species (ROS). Specifically *β*-carotene neutralize peroxyl radicals, leading to the formation of a carotenoid radical cation (CAR•+). This CAR• + can be reduced back to *β*-carotene by cellular antioxidants like tocopherol, thereby recycling *β*-carotene and reducing the propagation of lipid peroxidation (31). Carotenoids have anti-inflammatory properties, and increased serum concentrations can reduce the production of inflammatory mediators, potentially protecting telomeres from inflammatory damage (3). As potent antioxidants, they can neutralize free radicals and reduce oxidative stress, thus delaying telomere shortening in obese individuals (10, 32–34). Obesity-related unhealthy lifestyles, such as poor dietary habits and lack of exercise, may lead to reduced carotenoid intake, indirectly affecting telomere length. Hormonal fluctuations in obesity may also impact telomere length, and carotenoids may influence telomere length through hormone modulation or other signaling pathways (35).

For provitamin A carotenoids, including *α*-carotene, *β*-carotene, and *β*-cryptoxanthin, *β*-carotene, are abundantly found in yellow-orange fruits and green leafy vegetables. Notable sources of *β*-carotene include carrots, pumpkins, and celery (36). *β*-carotene may exhibit pro-oxidative properties at high concentrations or high oxygen partial pressures (10). The narrative review by Baliou et al. (2024) highlights the diverse benefits of the Mediterranean diet on telomere biology. It suggests that a diet rich in carotenoids from natural food sources may be more effective than supplementation in preserving telomere length, thereby helping to mitigate the progression of age-related diseases (37). *β*-carotene in adipose tissue may be metabolized into thrombotic or atherogenic derivatives, increasing the risk of cardiovascular disorders (38, 39). Conversely, non-provitamin A carotenoids, including lutein/zeaxanthin and trans-lycopene, have been reported to prevent DNA damage (40). Follow-up studies are needed to determine if specific carotenoids differentially protect telomere length. Notably, several studies have indicated that *β*-carotene supplementation alone, particularly at high doses, is linked to adverse outcomes, including an increased risk of all-cause mortality and a higher likelihood of lung cancer among individual at elevated risk for this disease (41, 42). Therefore, for daily intake, a mixed consumption of various carotenoids is recommended to avoid excessive intake of any single carotenoid class (43). Carotenoids may help reduce the risk of telomere shortening in obese individuals, underscoring the importance of a balanced dietary approach. Such as the Mediterranean diet, which offers broader nutritional support compared to isolated supplementation.

However, our study has limitations. First and foremost, our study utilized only the 2001–2002 cycle of the NHANES database (13). Further research and periodic studies are necessary to validate our findings. Also, as a cross-sectional study, it only explores the relationship between serum carotenoids and telomere length in obese individuals without establishing causality. More longitudinal studies and intervention trials are needed to clarify these associations and explore differences across gender, age, and ethnic groups (16). Additionally, there is no precise definition of high carotenoid concentrations, which may introduce bias.

Despite these limitations, our study highlights the potential relationship between serum carotenoids and telomere length in obese individuals, suggesting that increasing carotenoid intake may help delay telomere shortening, cellular aging, and related diseases in this population.

## 5 Conclusion

In conclusion, this study suggests that serum *β*-carotene is linearly and positively associated with longer telomere length in overweight and obese U.S. populations. Compared with overweight participants, obese participants ingested more *β*-carotene better for delaying telomere shortening. While the potential role of other carotenoids in delaying aging cannot be denied, further confirmation through future prospective studies is needed.

## Data Availability

The raw data supporting the conclusions of this article will be made available by the authors, without undue reservation.

## Footnotes

^1^ https://wwwn.cdc.gov/nchs/NHANES/Default.aspx

^2^ http://cdc.gov/nchs/nhanes

^3^ http://www.R-project.org

^4^www.empowerstats.com, X&Y Solutions, Inc.
