# Supplementary material for: Relationship between serum carotenoids and telomere length in overweight or obese individuals
Source: Front Nutr. 2024 Nov 22;11:1479994. doi: 10.3389/fnut.2024.1479994 (PMC11620882; doi:10.3389/fnut.2024.1479994)
Supplement: Supplementary file 1 [file Table_1.DOCX]

**Supplementary Table 1** Relationship between serum carotenoids and telomere length in non-overweight and non-obese people

| Exposure | Model I | Model II | Model Ⅲ |
| --- | --- | --- | --- |
|  | β (95%CI) P-value | β (95%CI) P-value | β (95%CI) P-value |
| Alpha-carotene | 5.9 (0.3, 11.5) 0.040 | 5.3 (-0.5, 11.1) 0.073 | 5.2 (-0.6, 11.1) 0.080 |
| Quartile of alpha-carotene |  |  |  |
| Q1 | 0 | 0 | 0 |
| Q2 | -7.0 (-110.0, 96.0) 0.894 | 4.3 (-102.1, 110.7) 0.937 | -1.7 (-108.8, 105.3) 0.975 |
| Q3 | 53.7 (-48.9, 156.4) 0.305 | 65.0 (-43.5, 173.4) 0.240 | 62.8 (-47.1, 172.7) 0.263 |
| Q4 | 102.8 (-1.9, 207.5) 0.054 | 101.5 (-12.0, 215.0) 0.080 | 95.6 (-20.3, 211.5) 0.106 |
| P for trend | 0.028 | 0.043 | 0.056 |
| Beta-carotene (trans + cis) | 1.5 (-0.0, 3.0) 0.057 | 1.5 (-0.1, 3.1) 0.068 | 1.4 (-0.2, 3.0) 0.081 |
| Quartile of Beta-carotene (trans + cis) |  |  |  |
| Q1 | 0 | 0 | 0 |
| Q2 | -27.2 (-129.3, 74.9) 0.602 | -24.5 (-128.0, 79.0) 0.643 | -23.6 (-127.9, 80.6) 0.657 |
| Q3 | 56.0 (-47.9, 160.0) 0.291 | 62.2 (-44.1, 168.4) 0.252 | 55.8 (-52.0, 163.6) 0.311 |
| Q4 | 79.4 (-27.1, 185.9) 0.144 | 85.7 (-25.2, 196.5) 0.130 | 81.4 (-31.8, 194.5) 0.159 |
| P for trend | 0.067 | 0.060 | 0.082 |
| Beta-cryptoxanthin | 3.5 (-0.9, 7.8) 0.116 | 5.1 (0.4, 9.8) 0.032 | 4.7 (-0.0, 9.5) 0.052 |
| Quartile of Beta-cryptoxanthin |  |  |  |
| Q1 | 0 | 0 | 0 |
| Q2 | 3.7 (-98.7, 106.1) 0.943 | -10.0 (-113.1, 93.1) 0.849 | -19.0 (-122.7, 84.6) 0.719 |
| Q3 | 50.1 (-52.1, 152.3) 0.337 | 51.9 (-52.9, 156.8) 0.332 | 43.6 (-62.5, 149.7) 0.421 |
| Q4 | 52.8 (-49.4, 154.9) 0.312 | 75.0 (-33.8, 183.8) 0.177 | 62.5 (-48.9, 173.9) 0.272 |
| P for trend | 0.212 | 0.110 | 0.174 |
| Combined lutein/zeaxanthin | 1.0 (-3.0, 4.9) 0.627 | 1.0 (-3.1, 5.2) 0.623 | 0.6 (-3.6, 4.9) 0.768 |
| Quartile of Combined lutein/zeaxanthin |  |  |  |
| Q1 | 0 | 0 | 0 |
| Q2 | -2.3 (-104.1, 99.6) 0.965 | -2.9 (-106.1, 100.4) 0.957 | -9.2 (-112.8, 94.5) 0.862 |
| Q3 | 31.2 (-70.6, 133.1) 0.548 | 27.0 (-78.0, 131.9) 0.615 | 20.1 (-85.6, 125.7) 0.710 |
| Q4 | 40.8 (-62.8, 144.5) 0.440 | 49.7 (-60.9, 160.3) 0.379 | 36.0 (-76.5, 148.6) 0.530 |
| P for trend | 0.348 | 0.316 | 0.448 |
| Trans-lycopene | 1.3 (-2.1, 4.7) 0.452 | 0.7 (-2.8, 4.1) 0.706 | 0.3 (-3.2, 3.8) 0.856 |
| Quartile of Trans-lycopene |  |  |  |
| Q1 | 0 | 0 | 0 |
| Q2 | 40.0 (-63.4, 143.4) 0.449 | 29.8 (-75.4, 135.0) 0.579 | 22.7 (-83.5, 128.9) 0.675 |
| Q3 | 33.9 (-70.7, 138.5) 0.526 | 14.8 (-91.4, 121.0) 0.785 | 8.8 (-98.2, 115.9) 0.871 |
| Q4 | 66.7 (-38.2, 171.5) 0.213 | 49.9 (-57.3, 157.2) 0.361 | 40.1 (-68.8, 149.0) 0.470 |
| P for trend | 0.254 | 0.439 | 0.548 |

Model I adjust for: Age; Sex.
Model II adjust for: Age; Sex; Education; Race; PIR; BMI; Physical activity; Energy.
Model Ⅲ adjust for: Age; Sex; Education; Race; PIR; BMI; Physical activity; Energy; Congestive heart failure; Cancer or malignancy; Hypertension; Smoking; Drinking.
