# Supplementary material for: Relationship between serum carotenoids and telomere length in overweight or obese individuals
Source: Front Nutr. 2024 Nov 22;11:1479994. doi: 10.3389/fnut.2024.1479994 (PMC11620882; doi:10.3389/fnut.2024.1479994)
Supplement: Supplementary file 2 [file Table_2.DOCX]

| α-carotene |  | Telomere length | P for interaction |
| --- | --- | --- | --- |
|  | N | β (95%CI) P-value |  |
| **Sex** |  |  | 0.2465 |
| Male | 1171 | 1.0 (-6.7, 8.7) 0.798 |  |
| Female | 1182 | 8.4 (1.2, 15.6) 0.022 |  |
| **Education** |  |  |  |
| Less Than 9th Grade | 312 | 2.5 (-6.2, 11.2) 0.570 | 0.2314 |
| 9-11th Grade | 390 | 12.4 (-7.6, 32.3) 0.224 |  |
| High School Grad | 562 | 17.3 (0.2, 34.3) 0.048 |  |
| Some College | 626 | -2.1 (-13.4, 9.2) 0.719 |  |
| College Graduate | 463 | 4.6 (-4.5, 13.7) 0.325 |  |
| **Race** |  |  |  |
| Mexican American | 531 | 0.2 (-7.8, 8.2) 0.955 | 0.2276 |
| Other Hispanic | 92 | -20.7 (-55.5, 14.1) 0.247 |  |
| Non-Hispanic White | 1241 | 1.0 (-6.9, 9.0) 0.799 |  |
| Non-Hispanic Black | 434 | 20.3 (7.4, 33.2) 0.002 |  |
| Other Race | 55 | 8.7 (-54.5, 71.9) 0.790 |  |
| **Physical activity** |  |  |  |
| No aerobic activity | 594 | 11.3 (-0.7, 23.4) 0.065 | 0.5341 |
| Low level exercise | 1251 | 3.0 (-3.4, 9.4) 0.364 |  |
| Moderate level exercise | 352 | 5.0 (-10.7, 20.8) 0.530 |  |
| High level exercise | 156 | -6.6 (-41.0, 27.9) 0.710 |  |
| **Congestive heart failure** |  |  |  |
| Yes | 75 | -12.2 (-92.4, 68.0) 0.767 | 0.6785 |
| No | 2278 | 4.6 (-0.7, 9.9) 0.086 |  |
| **Cancer or malignancy** |  |  |  |
| Yes | 209 | 15.3 (-1.6, 32.3) 0.077 | 0.1627 |
| No | 2144 | 3.3 (-2.3, 8.9) 0.243 |  |
| **Hypertension** |  |  |  |
| No | 1294 | 3.0 (-3.8, 9.8) 0.388 | 0.0773 |
| Yes | 1059 | 8.7 (0.4, 17.0) 0.041 |  |
| **Smoking** |  |  |  |
| Yes | 1148 | 7.4 (-2.2, 17.0) 0.130 | 0.4469 |
| No | 1205 | 3.4 (-2.9, 9.7) 0.295 |  |
| **Drinking** |  |  |  |
| Yes | 1593 | 5.0 (-1.6, 11.6) 0.136 | 0.619 |
| No | 760 | 3.8 (-4.9, 12.5) 0.392 |  |

**Supplementary Table 2** Relationship between α-carotene and telomere length in different subgroups Adjust for: Age; Sex; Education; Race; PIR; BMI; Physical activity; Energy; Congestive heart failure; Cancer or malignancy; Hypertension; Smoking; Drinking
