# Supplementary material for: Relationship between serum carotenoids and telomere length in overweight or obese individuals
Source: Front Nutr. 2024 Nov 22;11:1479994. doi: 10.3389/fnut.2024.1479994 (PMC11620882; doi:10.3389/fnut.2024.1479994)
Supplement: Supplementary file 3 [file Table_3.DOCX]

| β-cryptoxanthin |  | Telomere length | P for interaction |
| --- | --- | --- | --- |
|  | N | β (95%CI) P-value |  |
| **Sex** |  |  |  |
| Male | 1171 | 4.7 (0.1, 9.4) 0.047 | 0.1572 |
| Female | 1182 | 0.1 (-3.5, 3.7) 0.962 |  |
| **Education** |  |  |  |
| Less Than 9th Grade | 312 | 1.7 (-3.6, 7.1) 0.519 | 0.2886 |
| 9-11th Grade | 390 | 7.7 (1.4, 14.0) 0.017 |  |
| High School Grad | 562 | 1.0 (-6.1, 8.2) 0.775 |  |
| Some College | 626 | -3.2 (-9.5, 3.2) 0.332 |  |
| College Graduate | 463 | 4.1 (-2.4, 10.6) 0.217 |  |
| **Race** |  |  |  |
| Mexican American | 531 | 0.8 (-2.6, 4.2) 0.640 | 0.7182 |
| Other Hispanic | 92 | -9.7 (-24.4, 4.9) 0.197 |  |
| Non-Hispanic White | 1241 | 1.2 (-4.6, 7.0) 0.676 |  |
| Non-Hispanic Black | 434 | 6.7 (-1.9, 15.4) 0.129 |  |
| Other Race | 55 | 12.3 (-1.0, 25.5) 0.079 |  |
| **Physical activity** |  |  |  |
| No aerobic activity | 594 | 1.6 (-4.3, 7.5) 0.591 | 0.896 |
| Low level exercise | 1251 | 2.4 (-1.2, 6.0) 0.187 |  |
| Moderate level exercise | 352 | 0.4 (-8.8, 9.6) 0.931 |  |
| High level exercise | 156 | 0.9 (-13.4, 15.2) 0.904 |  |
| **Congestive heart failure** |  |  |  |
| Yes | 75 | 14.3 (-10.2, 38.8) 0.259 | 0.4645 |
| No | 2278 | 1.7 (-1.1, 4.6) 0.234 |  |
| **Cancer or malignancy** |  |  |  |
| Yes | 209 | 2.0 (-12.1, 16.1) 0.781 | 0.9757 |
| No | 2144 | 1.9 (-1.0, 4.8) 0.192 |  |
| **Hypertension** |  |  |  |
| No | 1294 | -0.7 (-4.5, 3.2) 0.737 | 0.0128 |
| Yes | 1059 | 4.7 (0.5, 8.9) 0.028 |  |
| **Smoking** |  |  |  |
| Yes | 1148 | 4.2 (-0.8, 9.1) 0.101 | 0.1523 |
| No | 1205 | 0.8 (-2.6, 4.3) 0.638 |  |
| **Drinking** |  |  |  |
| Yes | 1593 | 3.4 (-0.3, 7.2) 0.073 | 0.2458 |
| No | 760 | 0.3 (-3.9, 4.5) 0.892 |  |

**Supplementary Table 3** Relationship between β- cryptoxanthin and telomere length in different subgroups Adjust for: Age; Sex; Education; Race; PIR; BMI; Physical activity; Energy; Congestive heart failure; Cancer or malignancy; Hypertension; Smoking; Drinking.
