# Supplementary material for: Relationship between serum carotenoids and telomere length in overweight or obese individuals
Source: Front Nutr. 2024 Nov 22;11:1479994. doi: 10.3389/fnut.2024.1479994 (PMC11620882; doi:10.3389/fnut.2024.1479994)
Supplement: Supplementary file 4 [file Table_4.DOCX]

**Supplementary Table 4** Relationship between Combined lutein/zeaxanthin and telomere length in different subgroups

| Combined lutein/zeaxanthin |  | Telomere length | P for interaction |
| --- | --- | --- | --- |
|  | N | β (95%CI) P-value |  |
| **Sex** |  |  |  |
| Male | 1171 | 3.0 (-1.1, 7.0) 0.151 | 0.2864 |
| Female | 1182 | -0.0 (-4.0, 4.0) 0.996 |  |
| **Education** |  |  |  |
| Less Than 9th Grade | 312 | 1.7 (-3.6, 7.1) 0.525 | 0.3017 |
| 9-11th Grade | 390 | 2.4 (-4.1, 8.9) 0.466 |  |
| High School Grad | 562 | 2.7 (-4.9, 10.3) 0.488 |  |
| Some College | 626 | -2.2 (-8.3, 3.9) 0.474 |  |
| College Graduate | 463 | 2.5 (-3.7, 8.6) 0.435 |  |
| **Race** |  |  |  |
| Mexican American | 531 | 1.6 (-3.1, 6.2) 0.511 | 0.2614 |
| Other Hispanic | 92 | -7.7 (-21.3, 5.8) 0.268 |  |
| Non-Hispanic White | 1241 | 1.1 (-3.3, 5.5) 0.626 |  |
| Non-Hispanic Black | 434 | 3.7 (-2.9, 10.2) 0.272 |  |
| Other Race | 55 | -1.6 (-23.6, 20.4) 0.887 |  |
| **Physical activity** |  |  |  |
| No aerobic activity | 594 | 4.4 (-1.5, 10.3) 0.140 | 0.6804 |
| Low level exercise | 1251 | 0.8 (-2.9, 4.6) 0.668 |  |
| Moderate level exercise | 352 | -2.9 (-10.7, 4.8) 0.460 |  |
| High level exercise | 156 | 1.6 (-9.9, 13.1) 0.788 |  |
| **Congestive heart failure** |  |  |  |
| Yes | 75 | 10.9 (-5.2, 27.0) 0.191 | 0.2307 |
| No | 2278 | 0.9 (-1.9, 3.8) 0.522 |  |
| **Cancer or malignancy** |  |  |  |
| Yes | 209 | 5.0 (-4.8, 14.9) 0.320 | 0.3775 |
| No | 2144 | 1.0 (-1.9, 4.0) 0.491 |  |
| **Hypertension** |  |  |  |
| No | 1294 | -1.3 (-5.3, 2.7) 0.521 | 0.0306 |
| Yes | 1059 | 3.6 (-0.4, 7.6) 0.077 |  |
| **Smoking** |  |  |  |
| Yes | 1148 | 2.7 (-1.7, 7.1) 0.234 | 0.2638 |
| No | 1205 | -0.0 (-3.8, 3.7) 0.986 |  |
| **Drinking** |  |  |  |
| Yes | 1593 | 0.6 (-2.9, 4.1) 0.726 | 0.7728 |
| No | 760 | 2.5 (-2.2, 7.2) 0.295 |  |

Adjust for: Age; Sex; Education; Race; PIR; BMI; Physical activity; Energy; Congestive heart failure; Cancer or malignancy; Hypertension; Smoking; Drinking
