# Supplementary material for: Relationship between serum carotenoids and telomere length in overweight or obese individuals
Source: Front Nutr. 2024 Nov 22;11:1479994. doi: 10.3389/fnut.2024.1479994 (PMC11620882; doi:10.3389/fnut.2024.1479994)
Supplement: Supplementary file 5 [file Table_5.DOCX]

**Supplementary Table 5** Relationship between Trans-lycopene and telomere length in different subgroups

| Trans-lycopene |  | Telomere length | P for interaction |
| --- | --- | --- | --- |
|  | N | β (95%CI) P-value |  |
| **Sex** |  |  |  |
| Male | 1171 | 3.2 (0.3, 6.1) 0.030 | 0.0601 |
| Female | 1182 | -0.5 (-3.7, 2.7) 0.768 |  |
| **Education** |  |  |  |
| Less Than 9th Grade | 312 | 4.0 (-1.8, 9.8) 0.174 | 0.5127 |
| 9-11th Grade | 390 | -0.9 (-5.8, 4.0) 0.733 |  |
| High School Grad | 562 | 2.7 (-2.1, 7.5) 0.270 |  |
| Some College | 626 | -0.0 (-4.1, 4.0) 0.983 |  |
| College Graduate | 463 | 3.1 (-1.7, 8.0) 0.208 |  |
| **Race** |  |  |  |
| Mexican American | 531 | 4.7 (0.5, 9.0) 0.029 | 0.1361 |
| Other Hispanic | 92 | -2.5 (-13.2, 8.3) 0.656 |  |
| Non-Hispanic White | 1241 | 2.7 (-0.3, 5.7) 0.074 |  |
| Non-Hispanic Black | 434 | -3.5 (-8.4, 1.4) 0.166 |  |
| Other Race | 55 | -16.3 (-33.4, 0.7) 0.069 |  |
| **Physical activity** |  |  |  |
| No aerobic activity | 594 | 1.9 (-2.6, 6.4) 0.416 | 0.7874 |
| Low level exercise | 1251 | 2.7 (-0.2, 5.6) 0.064 |  |
| Moderate level exercise | 352 | 0.2 (-5.3, 5.7) 0.944 |  |
| High level exercise | 156 | -4.3 (-13.3, 4.6) 0.347 |  |
| **Congestive heart failure** |  |  |  |
| Yes | 75 | -3.7 (-18.2, 10.8) 0.617 | 0.2999 |
| No | 2278 | 1.5 (-0.7, 3.6) 0.177 |  |
| **Cancer or malignancy** |  |  |  |
| Yes | 209 | 7.5 (-0.7, 15.6) 0.075 | 0.1176 |
| No | 2144 | 0.9 (-1.3, 3.1) 0.416 |  |
| **Hypertension** |  |  |  |
| No | 1294 | 1.8 (-1.0, 4.6) 0.217 | 0.3834 |
| Yes | 1059 | 0.9 (-2.4, 4.1) 0.609 |  |
| **Smoking** |  |  |  |
| Yes | 1148 | 0.7 (-2.3, 3.7) 0.652 | 0.8657 |
| No | 1205 | 2.1 (-0.9, 5.1) 0.165 |  |
| **Drinking** |  |  |  |
| Yes | 1593 | 2.3 (-0.3, 4.9) 0.084 | 0.1897 |
| No | 760 | -0.7 (-4.4, 2.9) 0.700 |  |

Adjust for: Age; Sex; Education; Race; PIR; BMI; Physical activity; Energy; Congestive heart failure; Cancer or malignancy; Hypertension; Smoking; Drinking
